# Conservation and valorization of Italian potato biodiversity through genotype-by-sequencing (GBS)

**DOI:** 10.3389/fpls.2025.1629306

**Published:** 2025-07-21

**Authors:** M. Martina, R. Natale, E. Vergnano, A. M. Milani, S. Biricolti, L. Andrenelli, E. Portis

**Affiliations:** ^1^ Department of Agricultural, Forest and Food Sciences (DISAFA) – Plant Genetics, University of Turin, Turin, Italy; ^2^ Department of Agricultural, Environmental, Food and Forestry Science and Technology (DAGRI), University of Florence, Florence, Italy; ^3^ Interdepartmental Service Centre for Agricultural Chemical and Industrial Biotechnologies (CIBIACI), University of Florence, Florence, Italy

**Keywords:** biodiversity, genotyping-by-sequencing (GBS), potato, local varieties, cultural heritage

## Abstract

**Introduction:**

Traditional crop varieties are vital for conserving agrobiodiversity, ensuring food security, and preserving cultural heritage. However, Italian potato (*Solanum tuberosum*) landraces, shaped by centuries of farmer selection and adaptation to diverse agroecological conditions, are increasingly threatened by genetic erosion and agricultural homogenization.

**Methods:**

To support their conservation, we collected 61 accessions from 22 traditional Italian landraces, documented their traditional culinary uses, and evaluated tuber skin and pulp color. Virus presence was assessed and sanitation protocols were applied. Genetic diversity was analyzed using genotyping-by-sequencing (GBS), and comparisons were made with a global reference panel of 106 genotypes.

**Results:**

GBS data revealed marked genetic differentiation among the Italian landraces, with three main genetic clusters showing internal structure and minimal overlap with commercial varieties. The Italian germplasm was clearly distinct from worldwide accessions, underlining its unique genetic identity.

**Discussion:**

The observed diversity highlights the potential of traditional landraces as a valuable resource for breeding programs aimed at enhancing resilience to climate change. This integrated approach fosters both agrobiodiversity conservation and the socio-economic development of marginal areas, offering a model for safeguarding traditional crop diversity worldwide.

## 1 Introduction

The conservation and valorization of traditional potato varieties (*Solanum tuberosum* L.) are essential not only for their historical and cultural significance but also for their contributions to agricultural sustainability and biodiversity (Brush, 1995; Tobin et al., 2018). Italy, renowned for its diverse climates and rich agricultural traditions, has long served as a repository of unique plant genetic resources (Vaccino et al., 2024). The introduction of potatoes to Europe after the Columbian Exchange brought both challenges and opportunities, influencing local agricultural practices. Initial introductions of *Solanum tuberosum* andigena, adapted to short-day conditions and high-altitude environments in the Andes, faced limited success in most of Europe due to unfavorable photoperiods and climate conditions. It was only with the arrival of Solanum tuberosum, better suited to European latitudes, that potatoes became a staple crop, particularly in northern regions.

Over time, intensive agricultural practices and the pursuit of higher yields have led to the widespread adoption of uniform, high-yielding cultivars, often at the expense of traditional landraces (Grun, 1990; Solomon-Blackburn and Barker, 2001; Spanoghe et al., 2022). These local varieties possess adaptability to local extreme conditions and distinctive organoleptic qualities, offering valuable traits that modern cultivars lack. The loss of these local varieties has contributed to a narrowing of the genetic base, which poses significant risks to food security and crop adaptability in changing environments (Wang et al., 2019; Jo et al., 2022; Salgotra and Chauhan, 2023).

Preserving traditional potato varieties is not merely a matter of historical interest but a strategic necessity for safeguarding genetic diversity, which is essential for breeding new cultivars capable of withstanding emerging biotic threats, such as novel pathogen strains, and abiotic stressors, like drought and temperature fluctuations driven by climate change. By integrating traditional knowledge with advanced genetic and biotechnological tools, researchers can harness the unique properties of local landraces to strengthen sustainable agricultural systems (Handayani et al., 2019). The cultural and economic benefits of preserving these varieties also extend to local communities, where traditional crops are often embedded in regional identities and culinary traditions. Programs focused on recovery and valorization contribute to rural development, promote agrobiodiversity, and encourage the production and consumption of high-quality, region-specific food products. Such initiatives align with the growing trends for sustainable and locally sourced produce, highlighting traditional agriculture’s role in contemporary food systems (Johns et al., 2013).

The Italian territory, with its mountainous regions and multiple microclimates, is home to potato varieties that have developed specific adaptations, such as tolerance to marginal conditions and unique flavor profiles. Notable examples include the violet-skinned ‘Viola Calabrese,’ the renowned ‘Rossa di Cetica’ from Tuscany, and the ‘Quarantina Bianca’ from Liguria (Ghiselli et al., 2001; Manzelli et al., 2010). These local varieties have been preserved over generations by dedicated farmers who have recognized their value beyond mere productivity. Conservation efforts include *in vitro* culture and virus elimination techniques to ensure healthy propagation. The Department of Agricultural, Food, Environmental, and Forestry Sciences (DAGRI) at the University of Florence (Italy), has been pivotal in recovering and maintaining a collection of these landraces since the late 1990s (Angelini, 1999; Vecchio et al., 2007). Together with conservation techniques, molecular markers have been widely applied for the characterization of genetic diversity within and among these local varieties (Portis et al., 2005; Mauro et al., 2009; Carputo et al., 2013; Emanuelli et al., 2013; Vilanova et al., 2014). Indeed, compared to classical markers (i.e. SSRs), Next Generation Sequencing (NGS) technologies provide higher marker resolution, allowing higher precision in genetic and genomic analyses (Barchi et al., 2019; Saidi and Hajibarat, 2020; Toppino et al., 2020; Tripodi et al., 2021; Martina et al., 2022; Barchi et al., 2023; Martina et al., 2023, 2024). In recent years, genotype-by-sequencing (GBS) has become a key tool in potato genomics for studying genetic diversity, population structure, and trait mapping in both cultivated and wild germplasm. Applications include association studies for disease resistance traits (Sood et al., 2023) and the exploration of genome-wide variation to support conservation strategies and pre-breeding (Endelman et al., 2024; Sharma et al., 2024; Schilling et al., 2024). These studies underscore the power of GBS in revealing the complexity and richness of potato genetic resources.

We explored the genetic diversity of 61 accessions from 22 traditional potato varieties/ecotypes from Italy, aiming to collect, sanitize them from virus infection, preserve them *in vitro*, and finally return them to local farmers as fresh material. Genotype-by-sequencing (GBS) analyses revealed substantial genetic differentiation within Italian germplasm, and a significant level of differentiation with a set of 106 genotypes from other regions of the world. This approach safeguards biodiversity and promotes eco-friendly farming practices, supporting environmental sustainability and consumer health. Investments in traditional varieties yield socio-economic dividends, empower local communities, create jobs, uphold cultural heritage, and support high-quality, locally adapted crops sought by consumers for their unique characteristics and traceable origins. This holistic strategy preserves traditional agricultural knowledge, ensuring that future generations benefit from a resilient, diverse, and sustainable food system.

## 2 Materials and methods

### 2.1 Plant material

**Figure 1:**
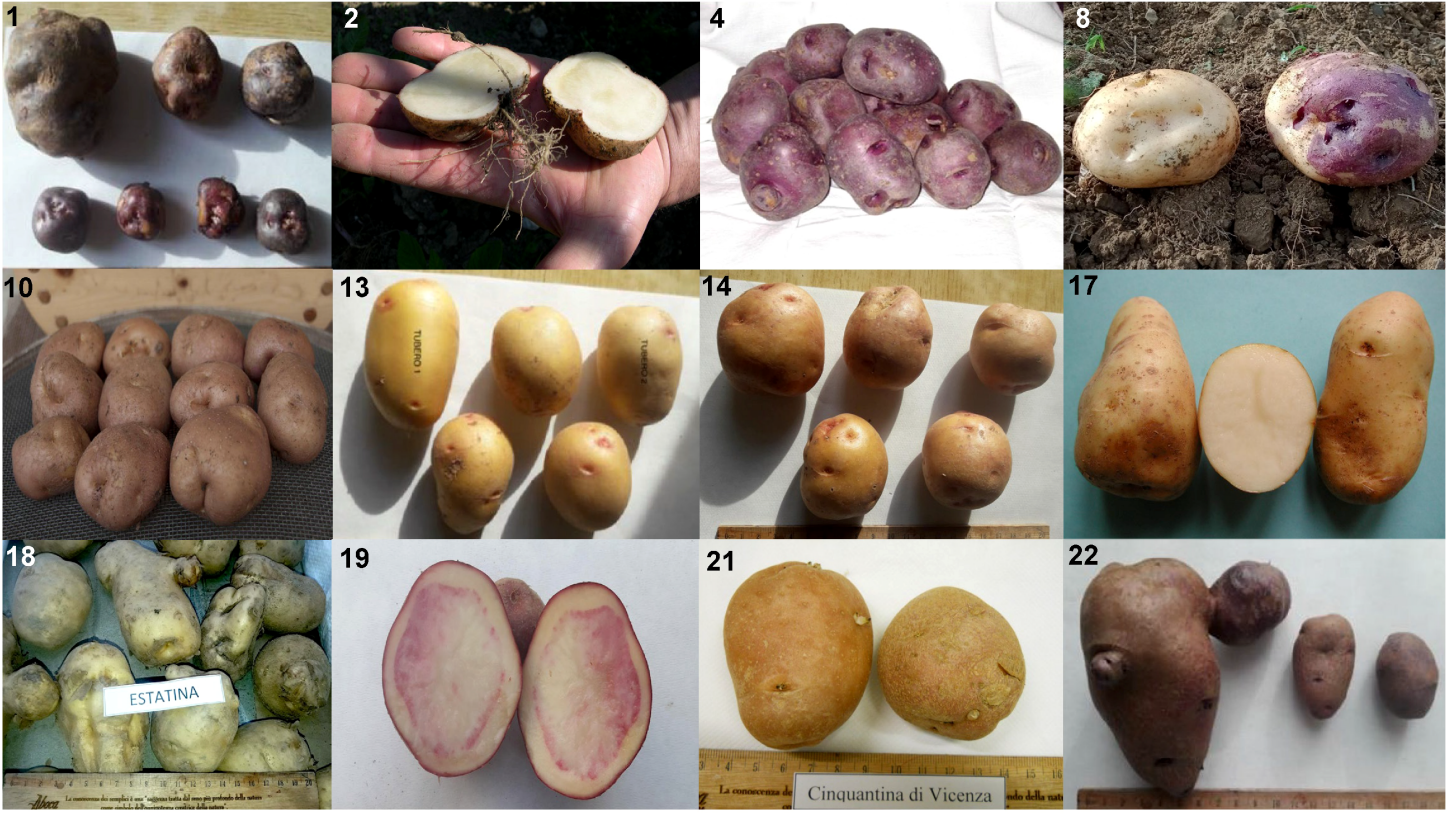
Example of the phenotypic variability within the analyzed collection (ID numbers refer to **Table 1**). From left to right, top to bottom: (1) Verrayes; (2) Fiocco di Neve; (4) Viola Calabrese; (8) Quarantina; (10) Formazza; (13) Occhi Rossi; (14) Rosa; (17) Castagno d’Andrea; (18) Estatina; (19) Rossa di Cetica; (21) Cinquantina Vicentina; (22) Mora della Reggenza.

A total of 61 accessions, representing twenty-two traditional Italian potato varieties/ecotypes, have been collected at the Department of Agriculture, Food, Environment, and Forestry (DAGRI) at the University of Florence (Italy) since the late 1990s (**Table 1**, **Figure 1**). Tubers were characterized for pulp and skin color according to the cultivated potato descriptors (Huaman et al., 1977), with minor modifications. On the collection day, thanks to the interaction with the custodian farmers, information on the traditional culinary destination was gathered. These varieties underwent an *in vitro* introduction process, virus testing, and virus elimination, following the methodology previously described (Andrenelli et al., 2002, 2018; Natale et al., 2024), as detailed in the next paragraph. Additionally, six commercial cultivars (Alba, Blu di San Gallo, Desiree, Institute de Beauvais, Kennebec, and Magenta) were included in the analysis as reference standards, based on previously detected genetic identities with some local accessions using microsatellite (SSR) markers (Mandolino et al., 2015). Furthermore, sequencing data from approximately 106 genotypes from various regions worldwide were retrieved from the NCBI database (PRJNA723185 - Sood et al., 2023) for comparative analysis.

**Table 1:** List of Italian local potato varieties/ecotypes (in bold) and selections from the DAGRI germplasm collection, including region and locality of Q30 provenance, number of accessions, and year of introduction.

Region of provenance	ID	Local variety/ecotype	Selection	Accessions (n°)	Year	Source
Aosta Valley	1	**Verrayes**	–	*3*	2022	Gressoney-Saint-Jean (AO)
Abruzzo	2	**Fiocco di neve**	–	*3*	2008	Farindola (PE)
	3	**Turchesa**	–	*2*	2002	Assergi (AQ)
Calabria	4	**Viola Calabrese**	*Germano*	*1*	1996	San Giovanni in Fiore (CS)
			*Rovale*	*1*		
Liguria	5	**Cannellina Nera**	–	*1*	1999	Val Graveglia (GE)
	6	**Giana Riunda**	*Mele*	*2*	2023	Mele (GE)
			*Montoggio*	*2*		Montoggio (GE)
	7	**Morella**	*Villa Rocca*	*1*	2019	Rezzoaglio (GE)
			*Bianca*	*1*		
	8	**Quarantina**	*Bianca*	*1*	1999	Valle Scrivia (GE)
			*Prugnona*	*1*		Val d’Aveto (GE)
	9	**Unica**	–	*1*	2019	Rezzoaglio (GE)
Piedmont	10	**Formazza**	–	*4*	2011	Formazza (VCO)
	11	**Morella della Valsesia**	–	*2*	2022	Alagna Valsesia (VC)
	12	**Piatlina**	–	*1*	2013	Monterosso Grana (CN)
	13	**Occhi rossi**	*Ambiel*	*1*	2022	Val Formazza (VCO)
			*Roti Oigje*	*6*	2016	
	14	**Rosa**	–	*2*	2022	Val Formazza (VCO)
	15	**Salecchio**	–	*1*	2023	Val Formazza (VCO)
	16	**Walser**	–	*2*	2016	Formazza (VCO)
Tuscany	17	**Castagno d’Andrea**	–	*3*	2002	Castagno d’Andrea (FI)
	18	**Estatina**	–	*5*	2013	Moncioni Bucine (AR)
	19	**Rossa di Cetica**	*Borghini*	*1*	1999	Castel San Nicolò (AR)
			*Poerio*	*2*		
	20	**Rossa di Sulcina**	–	*3*	2009	Sulcina (LU)
Veneto	21	**Cinquantina Vicentina**	–	*2*	2011	Vicenza (VI)
	22	**Mora della Reggenza**	*Cantele*	*2*	2023	Asiago (VI)
			*Rigoni*	*2*		
			*Muraro*	*1*		
			*Vellar*	*1*		

When a local variety was represented by more than one sample, the accession was identified by the “Selection” name. In the cases in which only one accession was included, no Selection name was attributed.

### 2.2 *In vitro* introduction and virus eradication protocol

**Figure 2:**
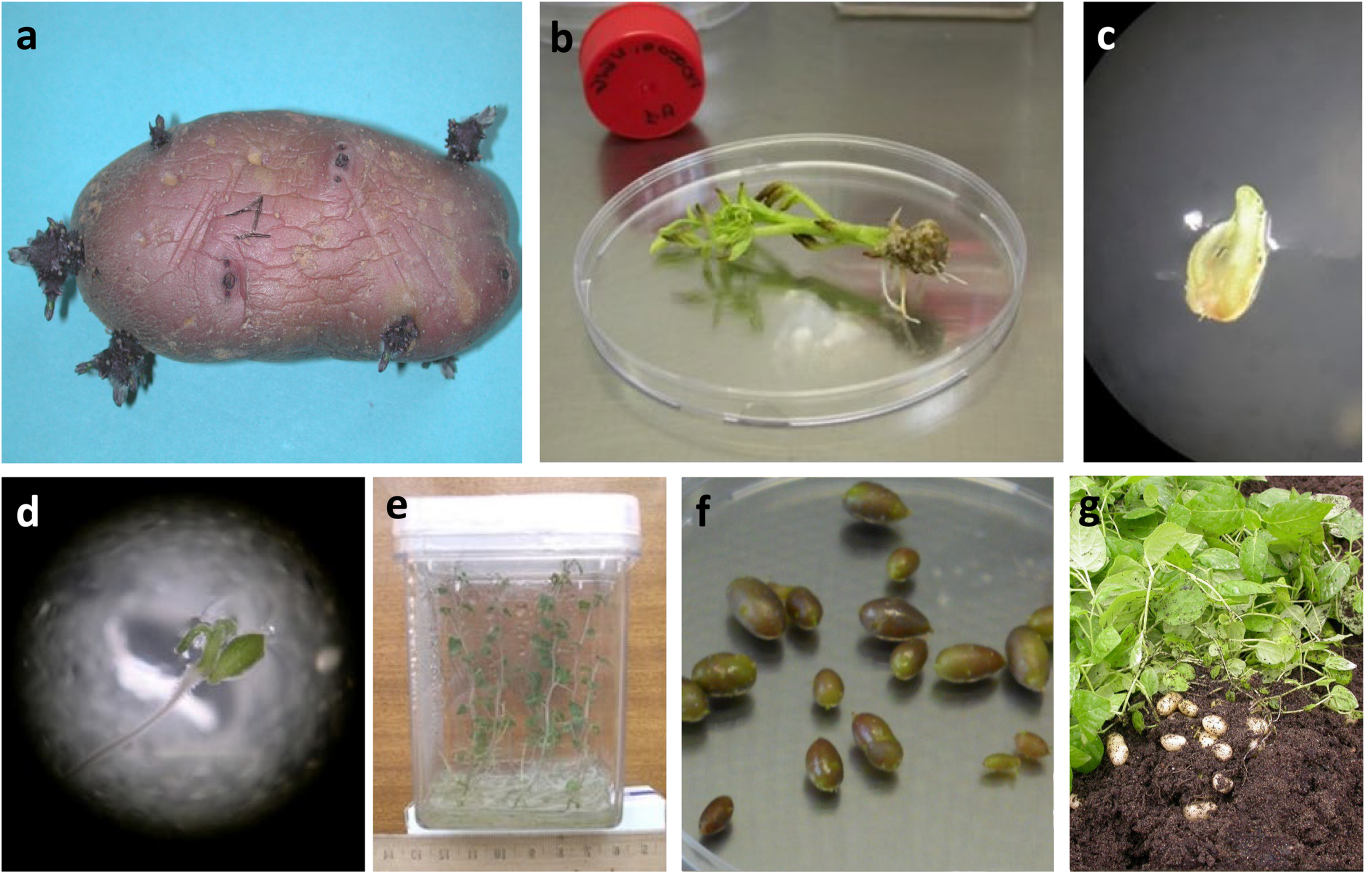
Main stages from *in vitro* introduction to seed tuber production: **(a)** tuber sprouting; **(b)** *in vitro* propagated plantets, obtained from tuber sprouts; **(c)** apical meristem growing on specific substrate; **(d)** apical meristem 20 days after explantation with root and leaf in initial differentiation; **(e)** *in vitro* virus free tested plantlets; **(f)** microtubers developed *in vitro*; g) vitro plant transplanted *in vivo*, showing minitubers production.

Collected tubers were carefully washed and allowed to sprout. Sprouts measuring 1–2 cm were harvested and disinfected by soaking in 70% ethanol for 30 seconds, followed by immersion in 2.5% sodium hypochlorite for 15 minutes. After multiple rinses with sterilized water, the sprouts were transferred to tubes containing Murashige and Skoog (MS) medium (Murashige and Skoog, 1962) supplemented with 25 g L^-1^ sucrose and 2 g L^-1^ Phytagel for growth and propagation (**Figure 1**). All varieties underwent virus molecular detection testing (RT-PCR) for the main potato viruses (PVX, PVY, PVS, PLRV) according to Mortimer-Jones et al. (2009), with minor modifications. The analyses were carried out by the “Regional Phytosanitary Service” of the Tuscany region, in Italy, which exercises control and official supervision over the phytosanitary status of plants and their products during cultivation, storage, and marketing stages, to verify the possible presence of harmful organisms to plants. Those testing positive were subjected to a sanitization protocol. Two weeks-old *in vitro* plants were placed in a climate chamber for thermotherapy treatment, following a one-month program: 4 hours at 35°C (daylight) and 4 hours at 30°C (darkness - Griffiths et al., 1990). After this period, the apical meristem (0.1 to 0.4 mm in size) of each plant was excised and cultured on a modified MS medium containing 0.1 mg L^-1^ gibberellic acid (GA_3_) and 0.04 mg L^-1^ kinetin to stimulate root development and growth. The regenerated plants were subsequently tested to confirm virus eradication. The complete process is illustrated in **Figure 2**.

### 2.3 DNA extraction, GBS-library preparation and sequencing

Genomic DNA was isolated from *in vitro* plantlets using a modified CTAB method (Doyle, 1991; Li et al., 2024). The purified DNA was resuspended in 100 µL of deionized water. To remove residual RNA, DNase-free RNase was added to the sample and incubated at 35°C for one hour. DNA concentration and purity were assessed using a Qubit™ 2.0 Fluorometer and by measuring absorbance ratios at 260/280 nm and 260/230 nm with a NanoDrop™ 2000 spectrophotometer. The integrity and size distribution of the genomic DNA were evaluated by agarose gel electrophoresis. Finally, samples were digested with MseI and EcoRI restriction enzymes to assess their compatibility with subsequent library construction and sequencing.

For genotyping-by-sequencing (GBS), libraries were prepared using a dual-enzyme strategy, following a modified version of the protocol by Poland et al., 2012. Genome complexity was reduced using the restriction enzymes *MseI* and *EcoRI*, and the resulting libraries were sequenced on an Illumina NovaSeq X system at the Biotechnology Center of the University of Wisconsin-Madison.

### 2.4 Variants calling, filtering and phylogenic analysis

Sequencing data from both the Italian local varieties/ecotypes and the dataset from Sood et al., 2023 were quality-filtered using *fastp* (Chen et al., 2018) and aligned to the DM-13 Potato reference genome (v6.1; Pham et al., 2020) using the GATK pipeline (Poplin et al., 2018). After alignment, variants were filtered based on the following criteria: a minimum depth of 15 (min DP15), a minor allele frequency threshold of ≤0.5 (MAF), and a missing data rate of ≤0.05. Pruning was carried out with the *SNPrelate* R package (Zheng et al., 2012), using a linkage disequilibrium (LD) threshold of 0.1. The resulting set of filtered SNPs was then used for Neighbor-Joining phylogenetic analysis in MEGA11 software (Tamura et al., 2021) with the p-distance method.

## 3 Results and discussion

### 3.1 Ecotypes preservation, geographical distribution and their main characteristics

**Figure 3:**
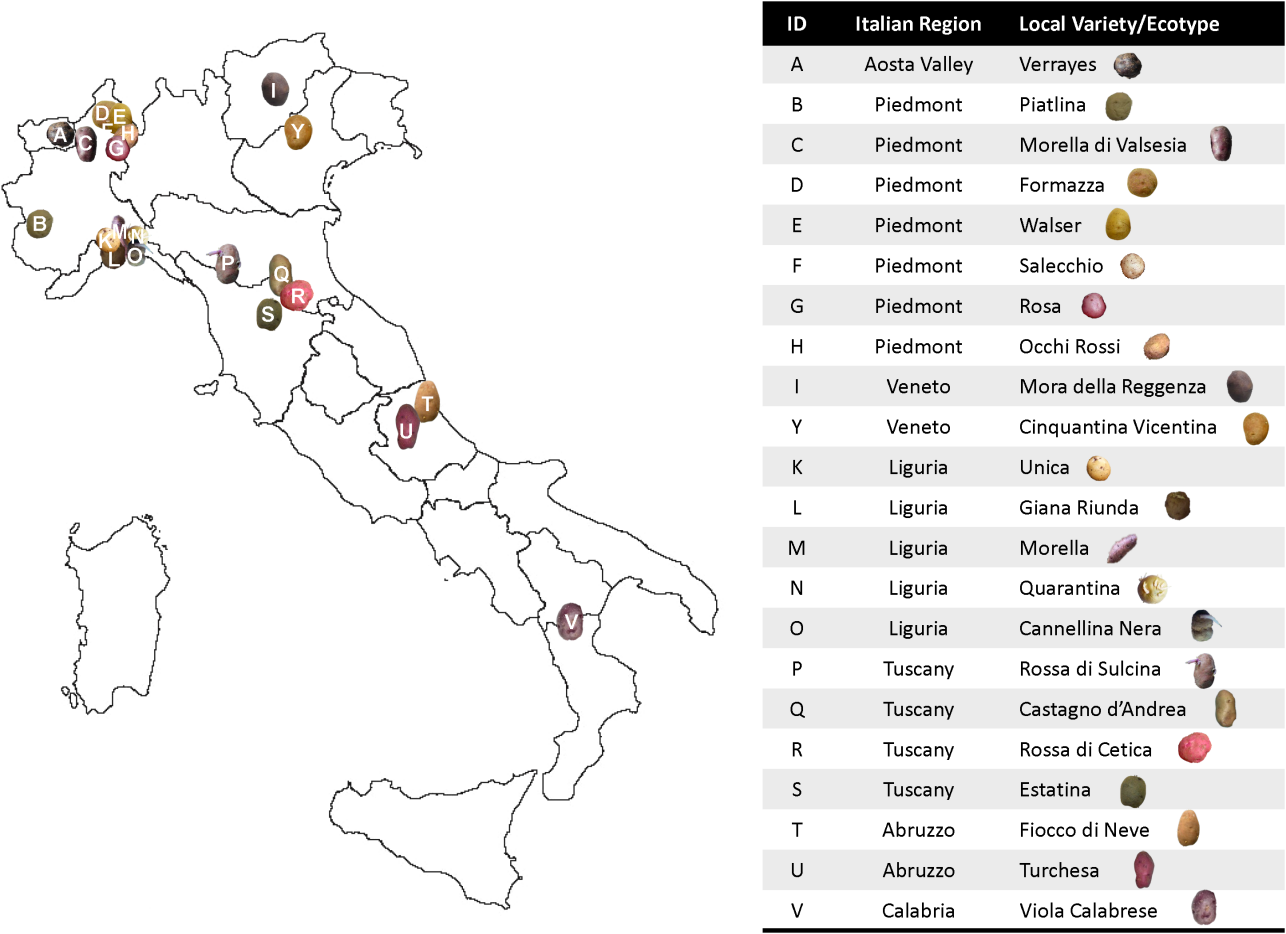
Distribution of the local varieties along the Italian peninsula.

Italy provides a favorable environment for the genetic diversification of potatoes, resulting in the selection of numerous local ecotypes adapted to the specific pedoclimatic conditions of its diverse regions (Perrino and Perrino, 2024). The geographic distribution analysis of these ecotypes reveals a predominance in the northern and central regions, with some varieties also present in southern Italy (**Figure 3**). In the northwestern Alps, particularly in Valle d’Aosta and Piedmont, varieties such as *Piatlina*, *Walser*, *Rosa*, and *Morella della Valsesia* exhibit good adaptability to mountain environments. These varieties produce medium-small tubers, with skin colors ranging from yellow to red. In Veneto region, *Mora della Reggenza* and *Cinquantina Vicentina* are traditionally cultivated in hilly and pre-alpine environments, with tubers having either dark or yellow skin (**Table 2**).

**Table 2:** List of Local varieties/ecotypes (in bold) along with their morphological characteristics and culinary uses.

ID	Local variety/ecotype	Skin color	Flesh color	Culinary destination
1	**Verrayes**	Purple, sometimes with orange mottles	Yellow	All purposes
2	**Fiocco di neve**	Yellow	White	All purposes
3	**Turchesa**	Purple	White	All purposes
4	**Viola Calabrese**	Purple	White	All purposes
5	**Cannellina Nera**	Yellow - brown with dark sprouts	White	Boiled, salad
6	**Giana Riunda**	Yellow	Yellow	All purposes
7	**Morella**	Purple with yellow mottles	White	All purposes
8	**Quarantina**	Yellow (sel. Bianca) Purple (sel. Prugnona)	White	All purposes
9	**Unica**	Purple	Yellow	All purposes
10	**Formazza**	Red	Yellow	“Gnocchi”
11	**Morella della Valsesia**	Purple, dark blue	White	All purposes
12	**Piatlina**	Yellow with purple eyes	White	All purposes
13	**Occhi rossi**	Yellow with red eyes	Yellow	All purposes
14	**Rosa**	Yellow with red eyes	Yellow	All purposes
15	**Salecchio**	Yellow	Yellow	All purposes
16	**Walser**	Yellow	Yellow	“Gnocchi”
17	**Castagno d’Andrea**	Yellow	White	All purposes
18	**Estatina**	Yellow	Yellow	All purposes
19	**Rossa di Cetica**	Red	White	“Gnocchi”
20	**Rossa di Sulcina**	Red	Yellow with red mottles	All purposes
21	**Cinquantina Vicentina**	Red	Yellow	All purposes
22	**Mora della Reggenza**	Purple, dark blue	White	All purposes

Along the Apennine ridge, Liguria region hosts ecotypes like *Quarantina* and *Giana Riunda*, while Tuscany showcases genetic diversity with varieties such as *Rossa di Cetica*, *Castagno d’Andrea*, and *Estatina*, possessing flesh colors that range from white to deep yellow. In the central-southern regions, *Fiocco di Neve* in Abruzzo and *Viola Calabrese* in Calabria feature skin colors ranging from pure white to deep purple.

Italian local potato ecotypes are distinguished not only by their geographic distribution but also by a range of agronomic and morphological characteristics. Many of these ecotypes have been selected over time for their resistance to harsh environmental conditions, such as poor soil, low temperatures, and seasonal drought. Mountain varieties tend to have shorter growth cycles and smaller tubers compared to those from temperate regions, which exhibit higher productivity and a greater variety of shapes and colors (**Table 2**). The preservation of these ecotypes by local farmers, particularly in extreme marginal areas like the upper *Valsesia* valley in Piedmont, is crucial. Here, the *Walser* population, who migrated from Switzerland at the end of the 14th century, selected varieties like *Formazza*, *Morella della Valsesia*, and *Verrayes* for their adaptability to extreme pedoclimatic conditions. These varieties became integral to the seasonal diet of the *Walser* community, composed of small farmers and shepherds. Preserving such genetic material is vital for maintaining genetic diversity, ensuring the resilience of potato crops to environmental changes, and safeguarding Italy’s rich agricultural heritage.

### 3.2 *In vitro* introduction and virus eradication

Viral infections pose a significant challenge to the conservation and cultivation of local potato landraces, often resulting in reduced yields and, in severe cases, the loss of valuable genetic material. Aiming at assessing the phytosanitary state of the Italian landraces, the collected material was screened at molecular level, revealing a widespread presence of PVX, PLRV, PVY, and PVS, with some ecotypes exhibiting multiple infections (**Table 3**).

**Table 3:** List of Local varieties/ecotypes (in bold) with the corresponding detected virus infection.

ID	Local variety/ecotype	Viruses test	ID	Local variety/ecotype	Viruses test
1	**Verrayes**	NEG	12	**Piatlina**	NEG
2	**Fiocco di neve**	NEG	13	**Occhi rossi**	NEG
3	**Turchesa**	NEG	14	**Rosa**	NEG
4	**Viola Calabrese**	PVX	15	**Salecchio**	PVX
5	**Cannellina Nera**	PVX, PLRV, PVY	16	**Walser**	NEG
6	**Giana Riunda**	PVX, PLRV, PVY	17	**Castagno d’Andrea**	NEG
7	**Morella**	PVX, PLRV, PVY	18	**Estatina**	NEG
8	**Quarantina**	PVX, PLRV, PVY	19	**Rossa di Cetica**	NEG
9	**Unica**	PVX, PLRV, PVY	20	**Rossa di Sulcina**	PVX, PVY, PVS, PLRV
10	**Formazza**	NEG	21	**Cinquantina Vicentina**	PVX
11	**Morella della Valsesia**	PVX, PLRV, PVY	22	**Mora della Reggenza**	PLRV, PVX

The “Viruses Test” column report the results of the virus assessment: the detected viruses are reported in the column, otherwise the landrace is score as Negative (NEG).

Notably, *Rossa di Cetica*, *Cannellina Nera*, *Giana Riunda*, and *Morella della Valsesia* were among the most affected, carrying several viral strains simultaneously. This widespread viral incidence underscores the challenges associated with the long-term vegetative propagation of traditional varieties without systematic phytosanitary management. To restore the health status of infected accessions, a sanitation protocol combining thermotherapy and meristem culture was applied. Post-treatment diagnostic tests confirmed the complete elimination of viral infections in all ecotypes, demonstrating the effectiveness of this method. These findings align with previous studies on the effectiveness of meristem culture in the recovery of vegetatively propagated crops (Wang et al., 2018; Bhat and Rao, 2020; Alavijeh et al., 2025). The availability of virus-free planting material is crucial for both conservation and sustainable seed tuber production. Healthy accessions not only preserve genetic diversity but also improve agronomic performance, particularly in low-input and organic farming systems (Tegen, 2016). Moreover, the recovery and maintenance of these local varieties play a key role in safeguarding Italians potato genetic heritage, facilitating their reintroduction into traditional cultivation systems. This effort is essential for maintaining culinary traditions, regional identity, and biodiversity, while also supporting the adaptation of landraces to changing environmental conditions.

### 3.3 Phylogenetic analysis

Overall, approximately 17 million reads (PE150) were generated, resulting in a total of about 56.5 gigabytes of raw data. The cleaned reads were aligned to the *Solanum tuberosum* reference genome (v6.1; Pham et al., 2020), leading to the identification of 8.3 million SNPs. After filtering, this number was reduced to 15.872.

**Figure 4:**
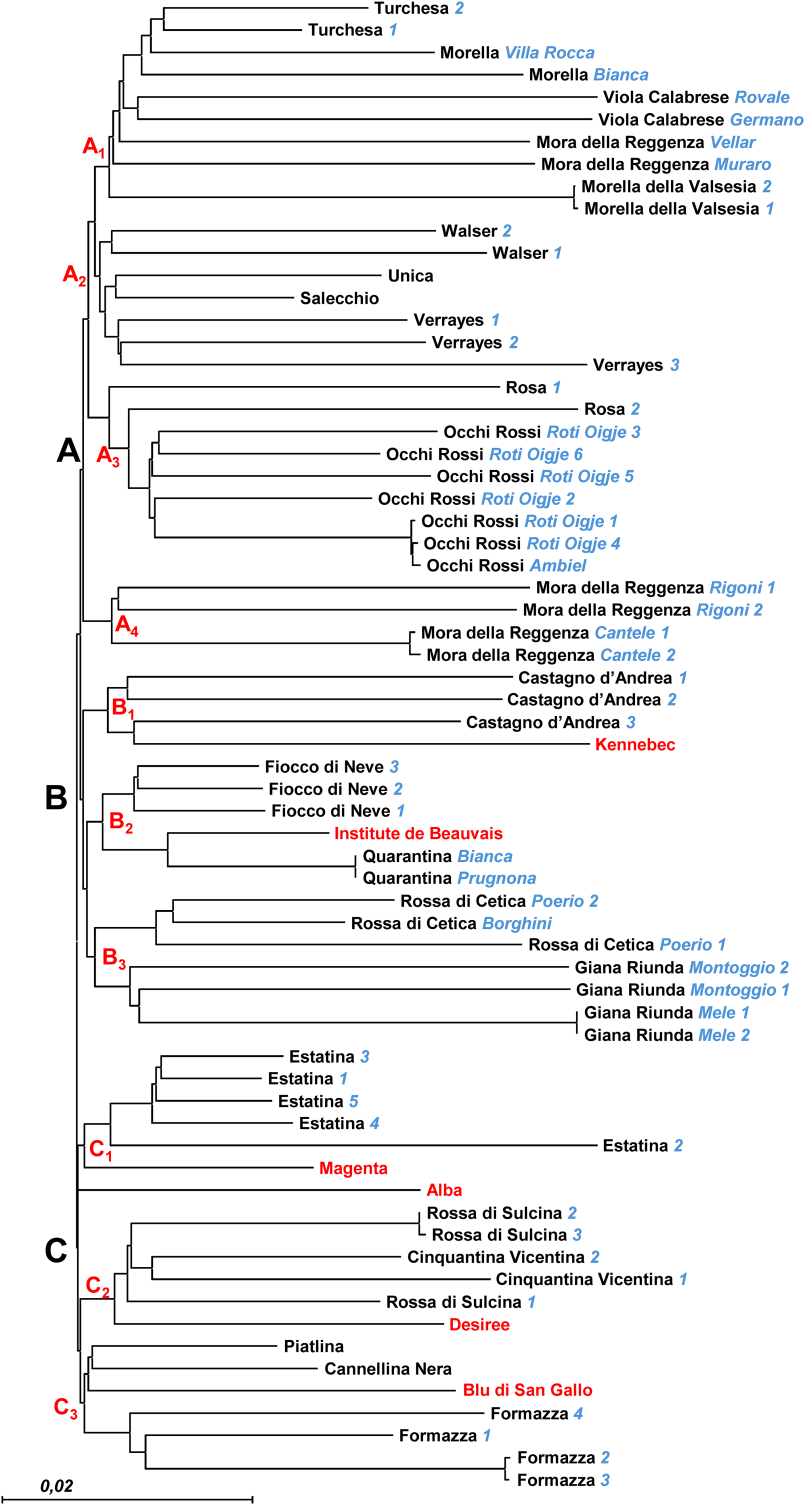
NJ dendrogram based on 15.872 SNP markers. Selection names and Accession numbers are indicated in blue. Reference commercial varieties are indicated in red. Three different clusters have been identified **(A–C)**, together with subclusters (in red).

The resulting NJ dendrogram (**Figure 4**) provides a distinct molecular fingerprint for each analyzed local variety/ecotype, organizing them into three main clusters (A, B, and C), each further divided into smaller subclusters (three or four per cluster). Although selections/provenances within each variety fall within the same subcluster, they are generally distinguishable, highlighting varying levels of within-variety diversity. This diversity reflects both genetic variation and potential influences from local adaptations or microenvironmental conditions. The five commercial varieties, used as reference material, are distributed across five different subclusters (B1, B2, C1, C2, and C3), each exhibiting a unique molecular profile. This contrasts with the findings of Mandolino et al., 2015, who used 12 SSR markers for molecular characterization of a small subset of the DAGRI collection and reported perfect identity between the SSR profiles of some commercial varieties and certain local varieties included in this analysis. This discrepancy highlights the enhanced resolution of the NGS-based approach employed in our study.

Branch A includes *Mora della Reggenza* (Vellar’ and ‘Muraro’ selections) and *Morella* accessions clustered together in A_1_, along with *Turchesa* and *Viola Calabrese*, suggesting a shared genetic background or conserved selection practices among these ecotypes. In contrast, the *Mora della Reggenza* ‘Rigoni’ and ‘Cantele’ selections are separated, clustering independently in A_4_ and showing clear genetic differentiation from other ecotypes with similar phenological traits. This differentiation could reflect distinct historical selection pressures or genetic drift specific to its location of origin. A_2_ comprises a group including *Unica*, *Salecchio*, and *Walser* accessions, along with the three *Verrayes* accessions, all characterized by a single molecular profile. This uniformity across multiple accessions may indicate a stable clonal lineage, possibly conserved due to its adaptability or favorable traits. A_3_ contains *Rosa* and *Occhi Rossi*, which, though grouped together, remain well distinguished from each other, suggesting that these varieties, while similar, have retained distinct genetic identities.

Branch B’s sub-cluster B_1_ confirms the previously detected similarity between *Castagno d’Andrea* and the commercial variety *Kennebec*, consistent with the findings of Mandolino et al. (2015). However, while the SSR analysis showed complete identity between these two varieties, the higher resolution of the NGS approach used here allows us to distinguish them. This finding emphasizes the sensitivity of NGS in revealing subtle genetic differences that SSR markers may overlook. Similarly, the relationship between the *Quarantina* ecotype and the commercial variety Institute de Beauvais, as well as with the *Fiocco di Neve* accessions in B_2_, was also observed by Mandolino et al. (2015), who nonetheless reported complete identity between *Quarantina* and the French-origin commercial variety. The distinctions observed here reinforce the potential of high-resolution markers to refine our understanding of genetic relationships. Finally, Branch B includes subcluster B_3_, where *Rossa di Cetica* and *Giana Riunda* selections are grouped together but remain distinguishable. This indicates that, while these selections may share some genetic similarities, they have diverged enough to maintain unique profiles, showcasing how local practices can introduce variability even within closely related selections.

In Branch C, the commercial varieties *Magenta* and *Alba* cluster in C_1_ with *Estatina* accessions, each retaining distinct profiles. This demonstrates that even when commercial and local varieties cluster together, they maintain unique molecular signatures, likely reflecting the influence of their respective breeding histories. In sub-cluster C_2_, the commercial variety *Desiree* clusters with *Rossa di Sulcina* and *Cinquantina Vicentina*, each maintaining distinctive molecular profiles. This contrasts with the findings of Mandolino et al. (2015), who reported complete identity among these varieties. Within this cluster, it is interesting to note that the *Rossa di Sulcina* accessions show significant internal variability, with two accessions exhibiting greater genetic distance from each other than from the *Cinquantina Vicentina* ecotype. This internal diversity could suggest either a broader genetic base within *Rossa di Sulcina* or the influence of localized environmental adaptation. Finally, the commercial variety *Blu di San Gallo* clusters with *Piatlina*, *Cannellina Nera*, and *Formazza*, with the *Formazza* accessions well-separated from the others in the final sub-cluster (C_3_) of **Figure 2**, underlining the diversity present even within this closely grouped set.

Notably, the resolution power of the NGS technique was sufficient to distinguish only a limited number of accessions sampled from the same provenance. For instance, this was the case for two accessions of *Morella della Valsesia* and *Giana Riunda* “Mele” selection, two of the three accessions of *Rossa di Sulcina*, and two of the four accessions of *Formazza*. Similarly, it could not distinguish between different provenances within the *Quarantina* ecotype, even though they are characterized by different skin colors. This phenotypic difference may be due to a point mutation not captured by our SNP marker dataset. The ability of the NGS technique to detect some but not all intraspecific variations highlight both its strengths and limitations in detecting subtle genetic differences within clonally propagated crops.

### 3.4 Comparison of the Italian germplasm with the world-wide accessions

Building on the work of Sood et al. (2023), we examined the genetic relationships between Italian potato local varieties/ecotypes and genotypes from around the world. In their study, the neighbor-joining (NJ) method was used to categorize 222 accessions and varieties into three main clusters (A, B, and C), which were further divided into subclusters. Their results indicated that, although no strict geographic pattern was evident, Indian varieties grown in the subtropical plains tended to cluster with a subset of andigena accessions, as well as accessions from Australia, New Zealand, and Afghanistan. In contrast, the other two clusters comprised a mix of varieties from Europe, North America, and South America, suggesting some level of geographic association with notable overlaps.

In our research, we compared a collection of Italian potato varieties with approximately 106 accessions from various parts of the world, as included in Sood et al. (2023 - **Supplementary Table 1**). Raw sequence reads were retrieved from the NCBI database (PRJNA723185), cleaned and aligned to the reference genome (Pham et al., 2020). SNP calling and filtering were then performed, yielding a set of 10,197 high-quality SNPs for further analysis. By integrating these global accessions with our Italian dataset, we aimed to explore whether Italian varieties would exhibit similar clustering patterns or if they would stand out genetically from other regional groups.

**Figure 5:**
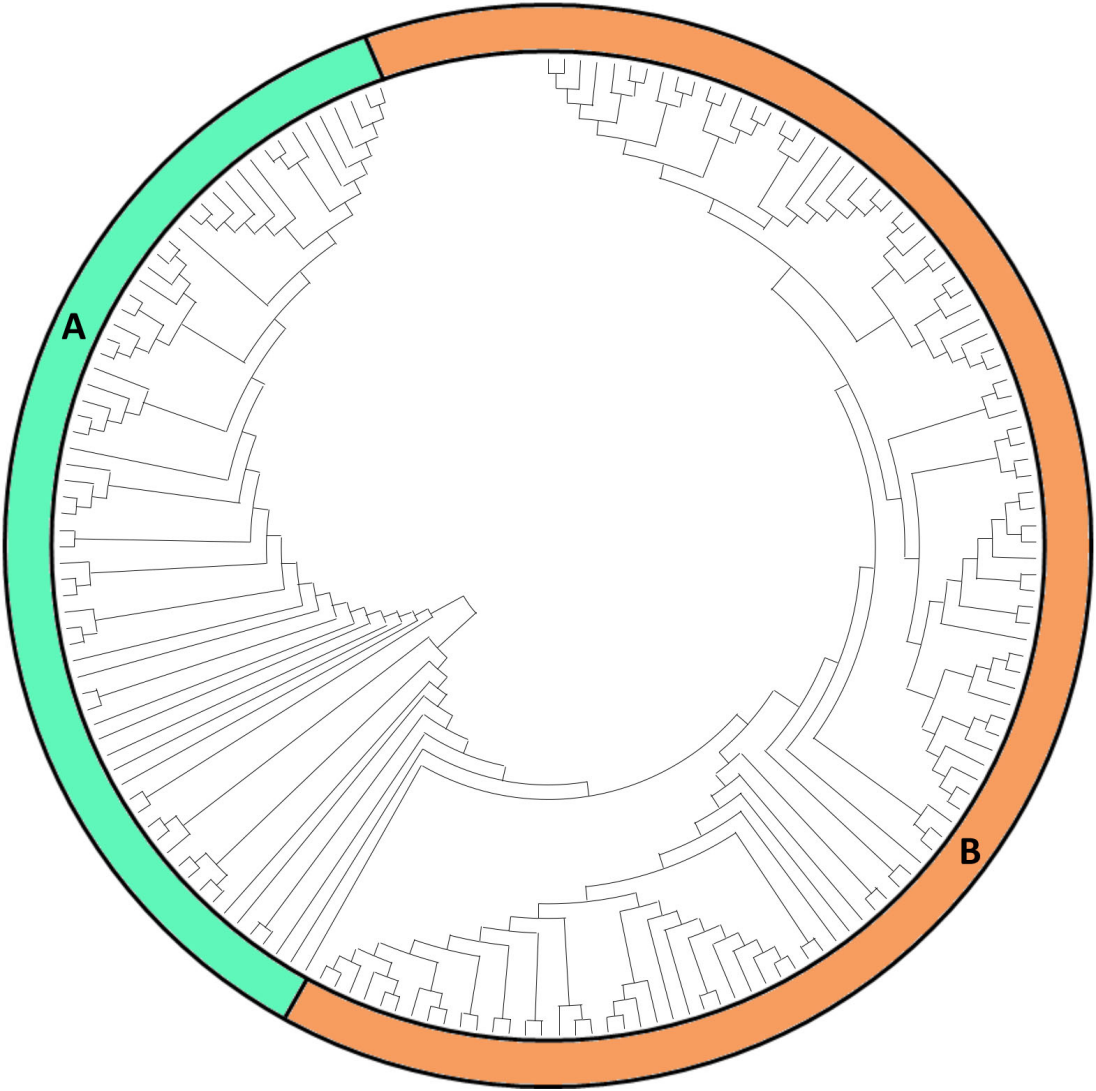
NJ dendrogram, constructed from 10,197 SNPs. Cluster **(A)** contains the accessions from Italy, while Cluster **(B)** contains the accessions from Sood et al., 2023.

The generated NJ dendrogram (**Figure 5**) reveals a marked separation between the Italian collection and the global accessions, highlighting a unique genetic signature among the Italian varieties. Unlike the findings of Sood et al. (2023), where some regional clustering patterns were observed but not strictly defined, our analysis demonstrates a clearer phylogenetic distinction. Italian varieties consistently formed a separate group, suggesting unique genetic characteristics likely shaped by local breeding practices and adaptation to regional conditions. This pronounced clustering of Italian varieties contrasts with the more intermixed clusters of international accessions, where European, North American, and South American varieties often appeared within the same groups, as reported by Sood et al. (2023).

Overall, our expanded approach, which combines Italian and international accessions, underscores a distinct phylogenetic identity for the Italian varieties. This separation may reflect historical, ecological, and selective pressures unique to Italian agricultural systems, offering valuable insights into the genetic diversity and potential breeding value of these local varieties within a global context.

## 4 Conclusions

The conservation and promotion of traditional potato varieties are crucial strategies for enhancing agricultural sustainability and socio-economic resilience.

In this study, we identified three major genetic clusters among 61 accessions representing 22 Italian landraces and observed a clear molecular separation from 106 international genotypes. Several landraces showed multiple viral infections, which were successfully eradicated through thermotherapy and meristem culture, enabling their recovery and safe propagation. These ecotypes, with their unique adaptations and qualities, provide invaluable genetic resources that can be harnessed to develop crops capable of facing current and future challenges, including climate change and the emergence of new pathogens. Preserving these landraces safeguards genetic diversity, ensuring a reservoir of traits that contributes to the development of resilient and productive cultivars. Moreover, the socio-economic benefits extend beyond agricultural improvements. By fostering the production and commercialization of traditional varieties, local communities can stimulate economic growth, create employment opportunities, and strengthen their cultural heritage. Initiatives that encourage collaboration among research institutions, local farmers, and policymakers, help build sustainable agricultural practices and regional networks that support the entire value chain. This, in turn, promotes rural development and ensures that high-quality, locally adapted food products reach a broader market.

The integration of traditional knowledge with modern scientific approaches is essential for maintaining biodiversity and empowering local farmers. Supporting programs that focus on the recovery, characterization, and utilization of traditional potato varieties ensure that the agricultural sector remains diverse and adaptable. These efforts not only address ecological and economic sustainability but also promote a deeper appreciation for the rich agricultural heritage and the potential of traditional crops to contribute to future food security. Investing in the conservation and valorization of traditional potato varieties is an investment in the future. It reinforces the importance of biodiversity as a foundation for sustainable development, creating a resilient agricultural system that benefits both present and future generations.

## Data Availability

Raw data supporting the conclusions of this article have been included in the present article. Sequencing data used in this study are openly available in the NCBI database (PRJNA1279639).
